# Baseline Alkaline Phosphatase Impacts Response Rates in Primary Biliary Cholangitis: Exploring Response to Elafibranor in ELATIVE

**DOI:** 10.1111/liv.70630

**Published:** 2026-04-06

**Authors:** Cynthia Levy, Christopher L. Bowlus, Eric Lawitz, Nuno Antunes, Benjamin Miller, Jianfen Shu, Claudia O. Zein, Kris V. Kowdley

**Affiliations:** ^1^ Schiff Center for Liver Diseases University of Miami Miami Florida USA; ^2^ Division of Digestive Health and Liver Diseases University of Miami School of Medicine Miami Florida USA; ^3^ Division of Gastroenterology and Hepatology UC Davis School of Medicine Sacramento California USA; ^4^ The Texas Liver Institute University of Texas Health San Antonio Texas USA; ^5^ Ipsen Cambridge Massachusetts USA; ^6^ Liver Institute Northwest Seattle WA USA

**Keywords:** alkaline phosphatase, biochemical markers, elafibranor, primary biliary cholangitis, prognosis

## Abstract

**Background & Aims:**

Baseline alkaline phosphatase (ALP) levels can influence the likelihood of achieving dichotomous biochemical response criteria in primary biliary cholangitis (PBC). This concept was explored using Week 52 data from the phase III ELATIVE trial (NCT04526665), which assessed elafibranor, a peroxisome proliferator‐activated receptor α/δ agonist approved as a second‐line treatment for PBC.

**Methods:**

Patients were grouped by baseline ALP. Outcomes assessed: biochemical response, ALP normalization, ALP change from baseline (CfB), transplant‐free survival (using GLOBE score).

**Results:**

At Week 52, biochemical response was achieved across all subgroups receiving elafibranor (≤ 2× upper limit of normal [ULN]: 86.7%, > 2–≤ 2.5× ULN: 80.0%, > 2.5–≤ 3× ULN: 52.0%, > 3–≤ 4× ULN: 22.2%, > 4× ULN: 18.8%), and one subgroup receiving placebo (≤ 2× ULN: 13.3%). ALP normalization occurred only with elafibranor (≤ 2× ULN: 53.3%, > 2–≤ 2.5× ULN: 12.0%, > 2.5–≤ 3× ULN: 12.0%, > 4× ULN: 12.5%). Mean ALP reductions were consistent across subgroups receiving elafibranor (overall CfB: −38.9%). Patients receiving elafibranor, regardless of biochemical response, had similar reductions in risk of liver transplant and/or liver‐related mortality within 15 years (−4.0% to −4.5%); among patients receiving placebo, reduced risk was only predicted in responders (−1.5%).

**Conclusions:**

In ELATIVE, lower baseline ALP correlated with higher biochemical response and ALP normalization rates with elafibranor. However, among patients receiving elafibranor, relative reductions in risk scores and ALP were consistent regardless of biochemical response and pre‐treatment ALP, indicating treatment benefit. Similar biochemical benefits were not observed among patients receiving placebo. These results support evaluating continuous measures and prognostic scores alongside dichotomous criteria to comprehensively assess efficacy.

**Trial Registration**: NCT04526665

## Key Points

- Elafibranor is a new treatment for PBC (primary biliary cholangitis), and levels of substances in the blood can indicate how someone with PBC is doing; reaching specific target levels shows the medicine is working, but people's starting levels of some substances, like ALP (alkaline phosphatase), can vary, which could influence whether they reach these targets.
- In the ELATIVE clinical trial, more people with low starting ALP reached a target called ‘biochemical response’ than those with high starting ALP; however, regardless of starting levels, everyone taking elafibranor showed similar improvements in ALP, and several people saw their levels return to normal.
- This research highlights that elafibranor is effective, but people with high starting ALP levels might not achieve biochemical response according to the definition used in the study, even if they are benefiting from treatment; hence, it is important to consider overall changes in substances and other factors to understand if treatment is working.

**Abbreviations**
ALP: alkaline phosphatase
ALT: alanine aminotransferase
AST: aspartate aminotransferase
CfB: change from baseline
GGT: gamma‐glutamyl transferase
LSM: liver stiffness measurement
PBC: primary biliary cholangitis
PPAR: peroxisome proliferator‐activated receptor
TB: total bilirubin
ULN: upper limit of normal

## 1 Introduction

Primary biliary cholangitis (PBC) is a rare, chronic, cholestatic liver disease characterized by immune‐mediated destruction of intrahepatic bile ducts, leading to cholestasis, liver fibrosis, and progressive liver damage [1, 2]. Elevated levels of alkaline phosphatase (ALP) and total bilirubin (TB) are indicators of cholestasis and are strongly associated with poor prognosis and increased risk of negative liver‐related outcomes [1, 3, 4, 5]. However, it is likely that patients with PBC have variable ALP levels.

While biochemical response criteria in PBC clinical trials often rely on dichotomous thresholds [6], such as a reduction in ALP and TB to predefined levels, this approach may overlook meaningful biochemical improvements, particularly in patients with higher baseline ALP levels. As these response thresholds are often defined as a multiple of the upper limit of normal (ULN), patients with a higher starting ALP value require a proportionally greater reduction to meet the same endpoint, potentially underestimating the treatment benefit in these individuals, given the near log‐linear association between ALP levels and transplant‐free survival [4]. Prognostic tools, such as GLOBE risk scores, incorporate continuous biochemical data and other variables to predict transplant‐free survival, and offer a more nuanced view of treatment benefit [7].

Elafibranor is a peroxisome proliferator‐activated receptor (PPAR)‐α/δ agonist approved as a second‐line treatment for PBC, which has demonstrated significant biochemical efficacy and a favourable safety profile in the phase III ELATIVE trial [8, 9, 10]. In ELATIVE, the primary endpoint of biochemical response at Week 52 utilized the POISE criteria, a dichotomous endpoint [10].

This analysis assessed the influence of baseline ALP levels on biochemical response rates, prognostic risk scores, and patient‐level changes in ALP over time in patients with PBC in the ELATIVE trial. A plain language summary of this article is provided in the Supporting Informations.

## 2 Methods

### 2.1 Trial Design

In ELATIVE (NCT04526665), a global, double‐blind, placebo‐controlled phase III trial, patients with PBC and an inadequate response or intolerance to ursodeoxycholic acid were randomized 2:1 to receive once‐daily elafibranor 80 mg or placebo for a minimum of 52 weeks [10]. Key inclusion criteria were an ALP level ≥ 1.67× ULN (174 U/L for females and 215 U/L for males) and a TB level ≤ 2× ULN (2.4 mg/dL) [10]. Two screening ALP measurements with ≤ 40% variability were required prior to randomization. The ELATIVE trial design and eligibility criteria have been reported in detail previously [10].

### 2.2 Study Endpoints

The primary endpoint was biochemical response at Week 52, defined as an ALP level < 1.67× ULN, with a reduction of ≥ 15% from baseline, and TB≤ULN [10]. ALP ULN values were 104 U/L in females and 129 U/L in males; TB ULN value was 1.2 mg/dL in females and males [10]. A key secondary endpoint was ALP normalization, defined as ALP≤ULN at Week 52 [10]. Mean percentage change from baseline to Week 52 in ALP was also assessed [10]. In this analysis, patients were categorized into subgroups based on their ALP level at baseline: ≤ 2× ULN, > 2–≤ 2.5× ULN, > 2.5–≤ 3× ULN, > 3–≤ 4× ULN, or > 4× ULN. Subgroups were chosen to achieve similar patient numbers, thereby ensuring that group sizes were meaningful and relevant for clinical interpretation.

Additionally, GLOBE scores were calculated at baseline and Week 52, and used to estimate transplant‐free survival rates at 5, 10, and 15 years in subgroups of patients grouped according to biochemical response (yes vs. no, defined by the primary endpoint), baseline ALP level (≤ 2× ULN, > 2–≤ 2.5× ULN, > 2.5–≤ 3× ULN, > 3–≤ 4× ULN, or > 4× ULN), and ALP normalization (yes vs. no).

### 2.3 Statistical Analysis

Biochemical response at Week 52 was analysed using a composite strategy; non‐response was imputed for patients with intercurrent events (trial regimen discontinuation or use of rescue therapy) before Week 52 [10]. For those with missing data, the nearest non‐missing assessment from the double‐blind period was used [10]. Statistical analysis methods have been detailed previously [10].

To determine the impact of elafibranor and placebo on estimated transplant‐free survival at 5, 10, and 15 years, the percentage difference in median risk between Week 52 and baseline, measured using GLOBE scores were calculated at these timepoints, with lower scores indicating better prognosis [7]. The formula used to calculate the GLOBE score at Week 52 is shown below: [7]

$$\begin{array}{c} \text{GLOBE}=0.044378\times \mathrm{age}\;\mathrm{at}\;\text{screening}+0.93982\\ \;\times \ln\left(\mathrm{TB}\times \mathrm{ULN}\;\mathrm{at}\;1\;\text{year follow}‐\mathrm{up}\right)\\ \;+0.335648\times \ln\left(\mathrm{ALP}\times \mathrm{ULN}\;\mathrm{at}\;1\;\text{year follow}‐\mathrm{up}\right)\\ \;-2.266708\times \text{albumin}\times \text{lower limit of normal}\;\mathrm{at}\;1\;\text{year follow}‐\mathrm{up}\\ \;-0.002581\times \text{platelet count}\;\mathrm{per}\;10^{9}/L\;\mathrm{at}\;1\;\text{year follow}‐\mathrm{up}+1.216865 \end{array}$$

## 3 Results

### 3.1 Patient Disposition and Baseline Characteristics

**FIGURE 1:**
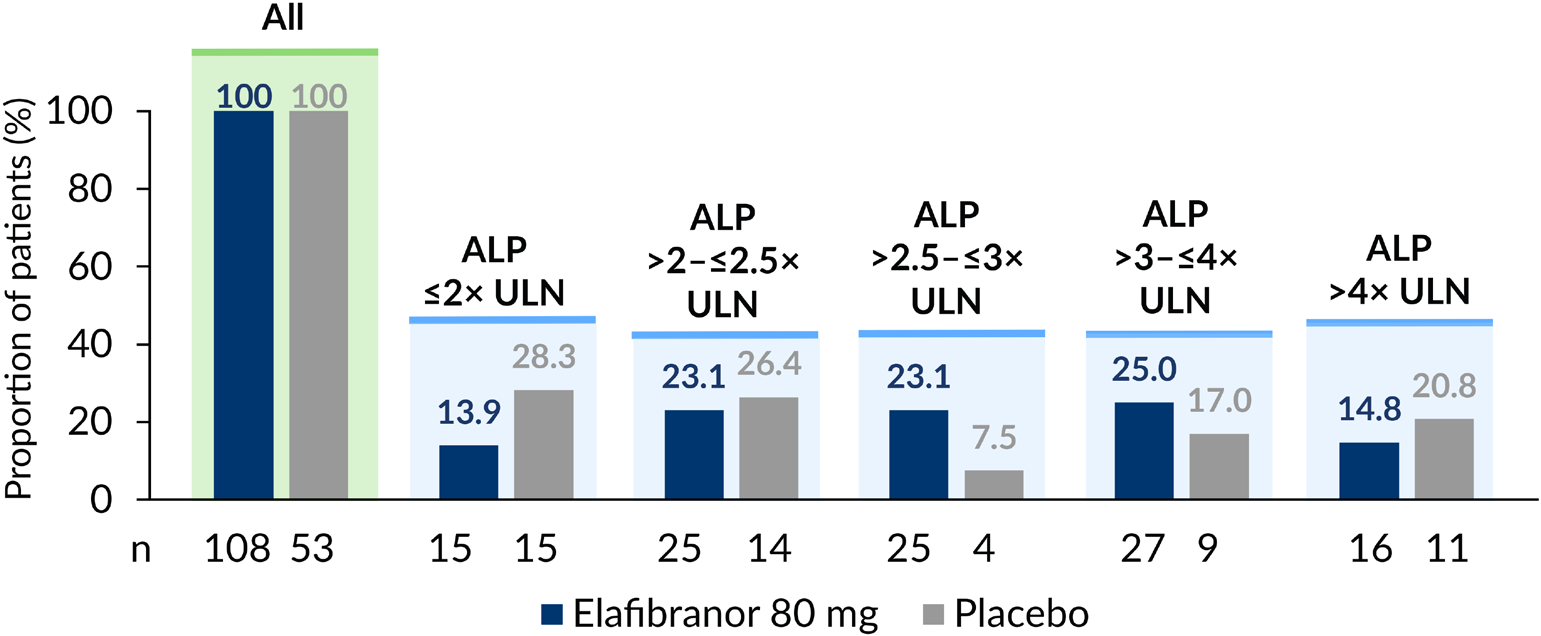
Proportion of patients in each ALP baseline subgroup across treatment arms. ALP: Alkaline phosphatase; ULN: Upper limit of normal.

A total of 161 patients were randomized to receive elafibranor (*n* = 108) or placebo (*n* = 53) [10]. Patients were evenly distributed across the baseline ALP subgroups: ≤ 2× ULN (*n* = 30), > 2–≤ 2.5× ULN (*n* = 39), > 2.5–≤ 3× ULN (*n* = 29), > 3–≤ 4× ULN (*n* = 36), and > 4× ULN (*n* = 27; Figure 1). Baseline demographics were similar across baseline ALP subgroups for both treatment arms, and the distributions of other biochemical markers of cholestasis at baseline were in line with the ALP stratification (Table 1). Across all patients, the mean ALP level at baseline was 321.9 (standard deviation [SD]: 150.9) U/L. [10] The minimum percentage reduction in ALP required to meet the ALP threshold component of the primary endpoint (< 1.67× ULN) and achieve ALP normalization was greater in the subgroup of patients with higher baseline ALP (Supplementary Table S1).

**TABLE 1:** Baseline demographics and disease characteristics of patients by ALP baseline subgroup.

Characteristic	Overall (*N* = 161)	ALP baseline subgroup				
		≤ 2× ULN (*n* = 30)	> 2–≤ 2.5× ULN (*n* = 39)	> 2.5–≤ 3× ULN (*n* = 29)	> 3–≤ 4× ULN (*n* = 36)	> 4× ULN (*n* = 27)
Age, years, mean (SD)	57.1 (8.7)	56.4 (9.3)	56.6 (9.6)	59.6 (6.7)	57.1 (9.3)	56.1 (7.9)
Sex, female, *n* (%)	154 (95.7)	29 (96.7)	36 (92.3)	27 (93.1)	35 (97.2)	27 (100)
Time since diagnosis, years, mean (SD)	8.0 (6.2)	7.9 (5.5)	6.4 (4.7)	8.0 (6.4)	9.1 (6.7)	9.2 (7.7)
ALP, U/L, mean (SD)	321.9 (150.9)	189.5 (19.8)	238.3 (24.0)	290.2 (22.7)	363.4 (33.7)	568.4 (198.7)
TB, mg/dL, mean (SD)	0.6 (0.3)	0.5 (0.2)	0.5 (0.2)	0.4 (0.1)	0.7 (0.3)	0.7 (0.4)
TB ≤ 0.6× ULN, *n* (%)	121 (75.2)	26 (86.7)	31 (79.5)	27 (93.1)	20 (55.6)	17 (63.0)
TB > 0.6× ULN, *n* (%)	40 (24.8)	4 (13.3)	8 (20.5)	2 (6.9)	16 (44.4)	10 (37.0)
AST, U/L, mean (SD)	45.7 (27.2)	29.9 (12.2)	34.2 (11.6)	37.8 (16.4)	56.3 (21.1)	74.3 (41.9)
ALT, U/L, mean (SD)	49.6 (32.6)	32.0 (16.1)	34.7 (15.0)	43.1 (30.5)	65.9 (33.7)	76.0 (39.6)
GGT, U/L, mean (SD)	215.5 (197.4)	117.3 (118.1)	159.2 (125.1)	144.0 (92.3)	276.1 (200.0)	402.1 (274.7)
Liver stiffness, kPa, mean (SD)	10.1 (8.2)^†^	7.8 (5.6)^‡^	10.1 (7.7)^§^	7.7 (2.1)^¶^	13.2 (11.6)^††^	11.2 (8.4)^¶^
LSM > 10.0 kPa and/or bridging fibrosis or cirrhosis on histology, *n* (%)	54 (35.1)^†^	5 (17.2)^‡^	13 (36.1)^§^	5 (18.5)^¶^	20 (57.1)^††^	11 (40.7)^¶^
LSM > 16.9 kPa and/or cirrhosis on histology, *n* (%)	16 (10.4)^†^	2 (6.9)^‡^	4 (11.1)^§^	0 (0.0)^¶^	6 (17.1)^††^	4 (14.8)^¶^

Abbreviations: ALP, alkaline phosphatase; ALT, alanine aminotransferase; AST, aspartate aminotransferase; GGT, gamma‐glutamyl transferase; LSM, liver stiffness measurement; SD, standard deviation; SEM, standard error of the mean; TB, total bilirubin; ULN, upper limit of normal.

^**†**^ *n* = 154.

^**‡**^ *n* = 29.

^**§**^ *n* = 36.

^¶^ *n* = 27.

^††^ *n* = 35.

A trend was observed where a larger proportion of patients in the higher ALP groups had advanced disease (defined as liver stiffness measurement [LSM] > 10.0 kPa and/or bridging fibrosis and/or cirrhosis on histology). Similarly, patients with higher baseline ALP also tended to have higher baseline levels of aspartate aminotransferase (AST), alanine aminotransferase (ALT), and gamma‐glutamyl transferase (GGT). Notably, TB levels did not differ substantially between the baseline ALP subgroups, and the majority of patients had TB ≤ 0.6× ULN across all subgroups.

### 3.2 Biochemical Response Rates at Week 52

**FIGURE 2:**
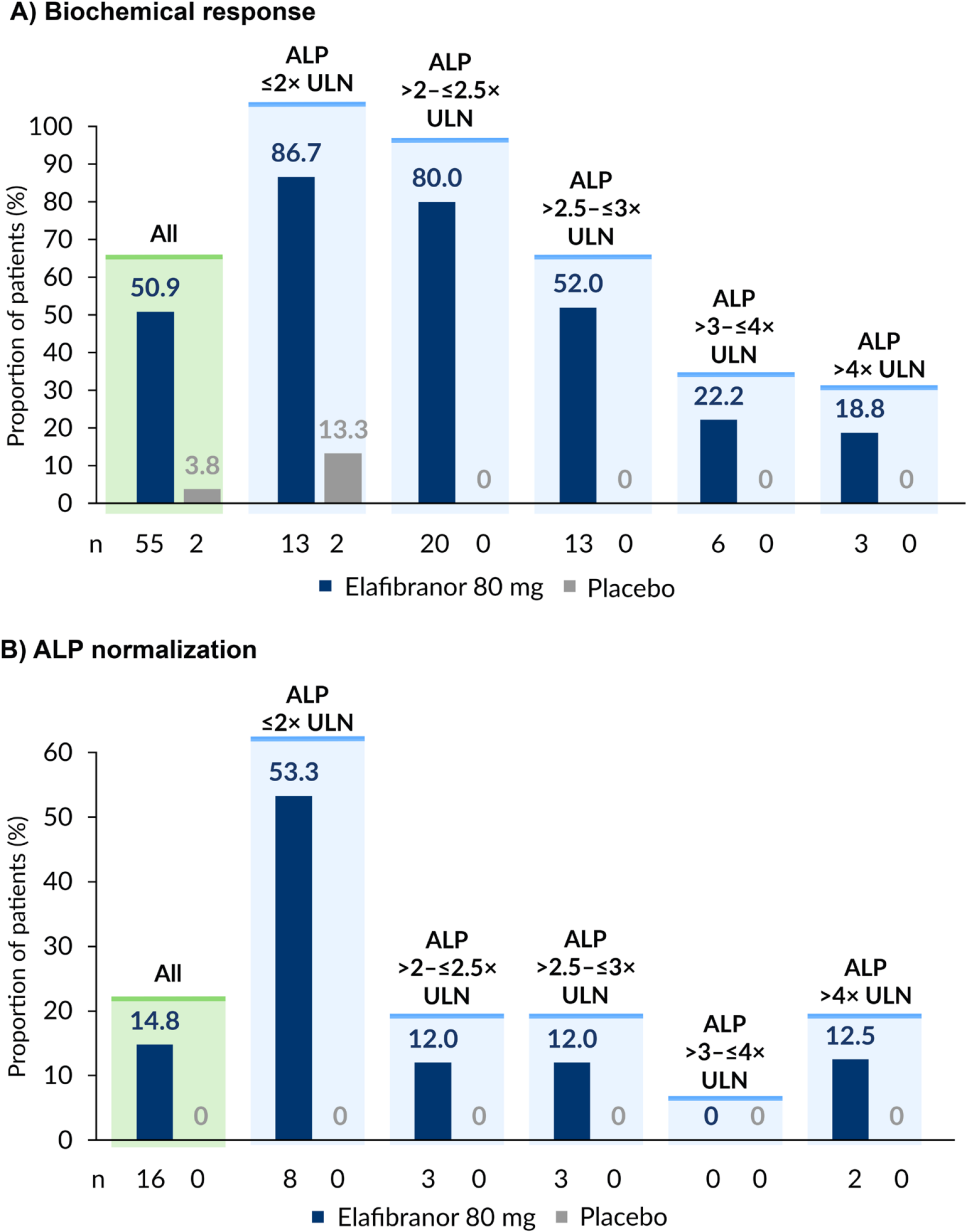
Proportion of patients achieving biochemical response^†^ and ALP normalization at Week 52, stratified by baseline ALP level. Percentages are expressed as a percentage of patients within the respective baseline ALP subgroups (All: Elafibranor *n* = 108, placebo *n* = 53; ALP ≤ 2× ULN: Elafibranor *n* = 15, placebo *n* = 15; ALP > 2–≤ 2.5× ULN: Elafibranor *n* = 25, placebo *n* = 14; ALP > 2.5–≤ 3× ULN: Elafibranor *n* = 25, placebo *n* = 4; ALP > 3–≤ 4× ULN: Elafibranor *n* = 27, placebo *n* = 9; ALP > 4× ULN: Elafibranor *n* = 16, placebo *n* = 11). ^†^According to the primary endpoint. ALP: Alkaline phosphatase; ULN: Upper limit of normal.

Overall, a biochemical response according to the primary endpoint was achieved by 50.9% (n/*N* = 55/108) of patients receiving elafibranor and 3.8% (n/*N* = 2/53) of patients receiving placebo (Figure 2A) [10]. Among patients receiving elafibranor, a biochemical response was observed in 86.7% (n/*N* = 13/15) of patients with baseline ALP ≤ 2× ULN, 80.0% (n/*N* = 20/25) of patients with baseline ALP > 2–≤ 2.5× ULN, 52.0% (n/*N* = 13/25) of patients with baseline ALP > 2.5–≤ 3× ULN, 22.2% (n/*N* = 6/27) of patients with baseline ALP > 3–≤ 4× ULN, and 18.8% (n/*N* = 3/16) of patients with baseline ALP > 4× ULN. A biochemical response was observed in 13.3% (n/*N* = 2/15) of patients with baseline ALP ≤ 2× ULN in the placebo treatment arm. No patients in the placebo treatment arms with baseline ALP > 2× ULN achieved biochemical response.

Only patients receiving elafibranor achieved ALP normalization at Week 52 (Figure 2B). Specifically, 53.3% (*n*/*N* = 8/15) of patients with baseline ALP ≤ 2× ULN, 12.0% (n/*N* = 3/25) of patients with baseline ALP > 2–≤ 2.5× ULN and ALP > 2.5–≤ 3× ULN, and 12.5% (*n*/*N* = 2/16) of patients with baseline ALP > 4× ULN achieved ALP normalization. The percentage reduction in ALP levels from baseline in the two patients who achieved ALP normalization with a baseline ALP > 4× ULN were −84.5% and −82.2%.

**FIGURE 3:**
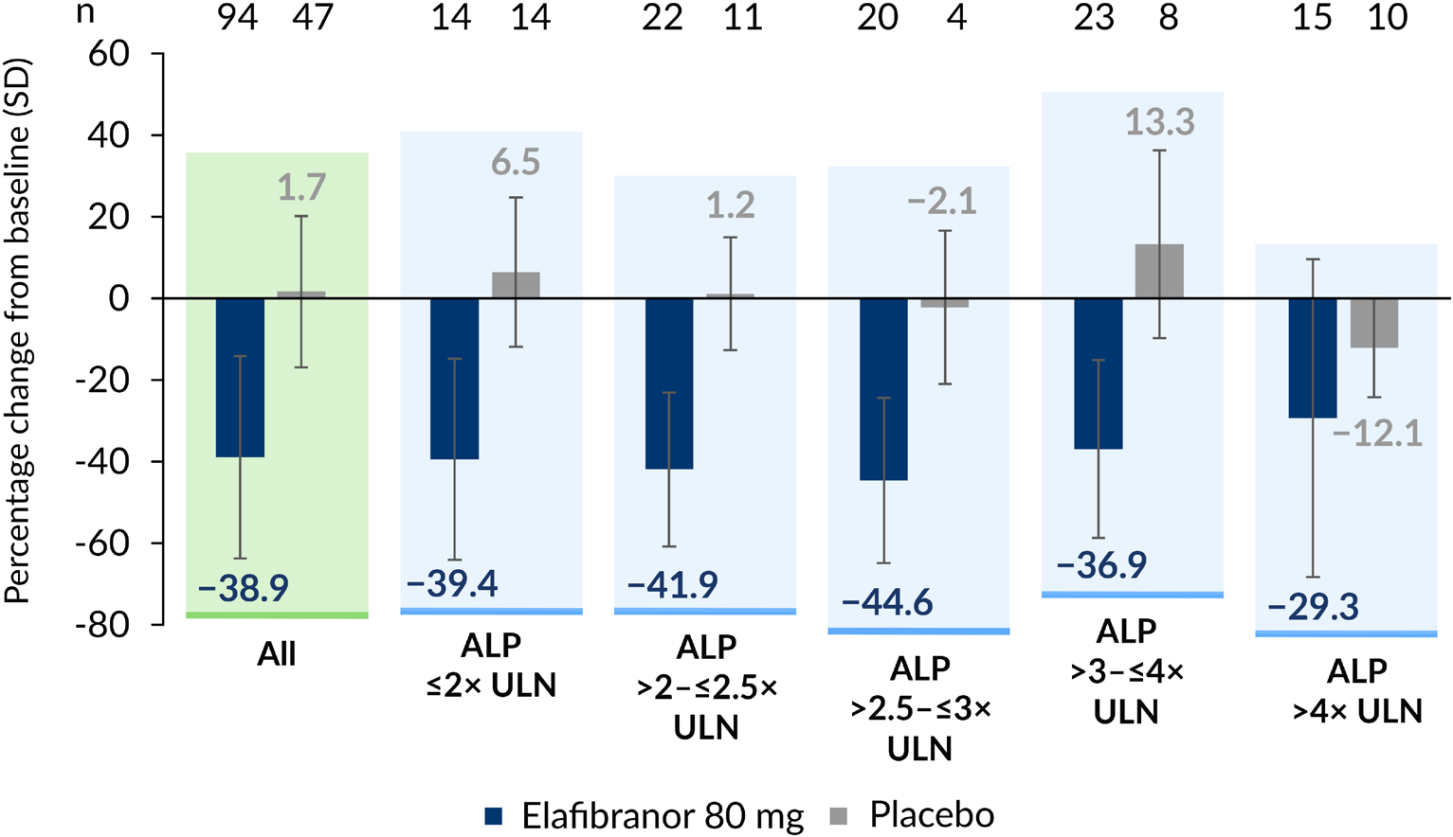
Mean percentage change from baseline in ALP at Week 52, stratified by baseline ALP level. ALP: Alkaline phosphatase; SD: Standard deviation; ULN: Upper limit of normal.

**FIGURE 4:**
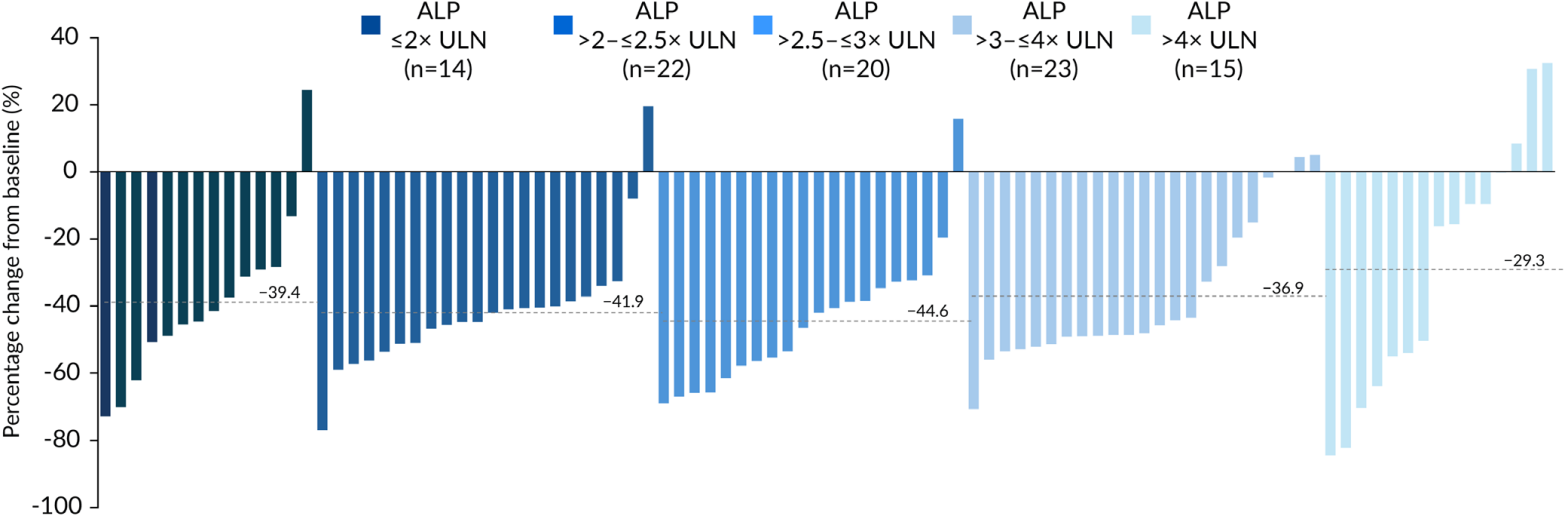
Waterfall plot of percentage change from baseline to Week 52 in ALP in patients receiving elafibranor stratified by baseline ALP levels. Dotted lines represent the mean percentage change from baseline in each baseline ALP subgroup. ALP: Alkaline phosphatase; ULN: Upper limit of normal.

The overall mean percentage change from baseline (SD) in ALP at Week 52 was –38.9% (24.8%) in patients receiving elafibranor compared with +1.7% (18.5%) in patients receiving placebo. Changes were variable in patients receiving placebo, and ALP increased in some subgroups: ALP ≤ 2× ULN (+ 6.5% [18.3%]), ALP > 2–≤ 2.5× ULN (+1.2% [13.8%]), ALP > 2.5–≤ 3× ULN (−2.1% [18.8%]), ALP > 3–≤ 4× ULN (+13.3% [23.0%]), ALP > 4× ULN (−12.1% [12.1%]). In contrast, substantial relative reductions in ALP were observed in patients receiving elafibranor across all ALP subgroups from baseline to Week 52, and reductions were generally consistent between the subgroups: ALP ≤ 2× ULN (−39.4% [24.7%]), ALP > 2–≤ 2.5× ULN (−41.9% [18.8%]), ALP > 2.5–≤ 3× ULN (−44.6% [20.2%]), ALP > 3–≤ 4× ULN (−36.9% [21.8%]), ALP > 4× ULN (–29.3% [39.0%]) (Figure 3). At Week 52, among patients receiving elafibranor with non‐missing data, 90.4% (n/*N* = 85/94) of patients had a reduction from baseline in ALP. Patient‐level changes in ALP from baseline to Week 52 in patients receiving elafibranor stratified by baseline ALP levels are presented in Figure 4. Exemplifying the impact of varying baseline ALP levels, 12 patients receiving elafibranor had ALP decreases of 40.0% or more at Week 52 and had TB≤ULN, yet were categorized as non‐responders because their ALP level did not meet the threshold of < 1.67× ULN.

### 3.3 Prognostic Risk Scores and Estimated Transplant‐Free Survival

Regardless of baseline ALP, patients receiving elafibranor had a reduced risk of requiring a liver transplant and/or liver‐related mortality at 5, 10, and 15 years, estimated based on GLOBE score (Supplementary Figure S1A). Risk reduction at 5, 10, and 15 years was observed in patients receiving elafibranor across all ALP subgroups. The greatest risk reductions were observed in patients in the ALP > 4× ULN subgroup. Patients in the ALP > 2.5–≤ 3× ULN subgroup showed a comparable risk reduction to those in the ALP > 4× ULN subgroup at 15 years (−6.2% vs. −5.5%). Patients receiving placebo had an increased predicted risk at 15 years in all ALP subgroups except the ALP > 2.5–≤ 3× ULN subgroup, where patients had a −0.8% reduction in median risk at 15 years (Supplementary Figure S1B). In patients receiving elafibranor, risk reduction was predicted regardless of ALP normalization; those who achieved ALP normalization had a greater decrease in median risk at 5, 10, and 15 years compared with patients who did not achieve ALP normalization (Supplementary Figure S2A). All patients receiving placebo had an increase in median risk from 5 to 15 years (Supplementary Figure S2B).

**FIGURE 5:**
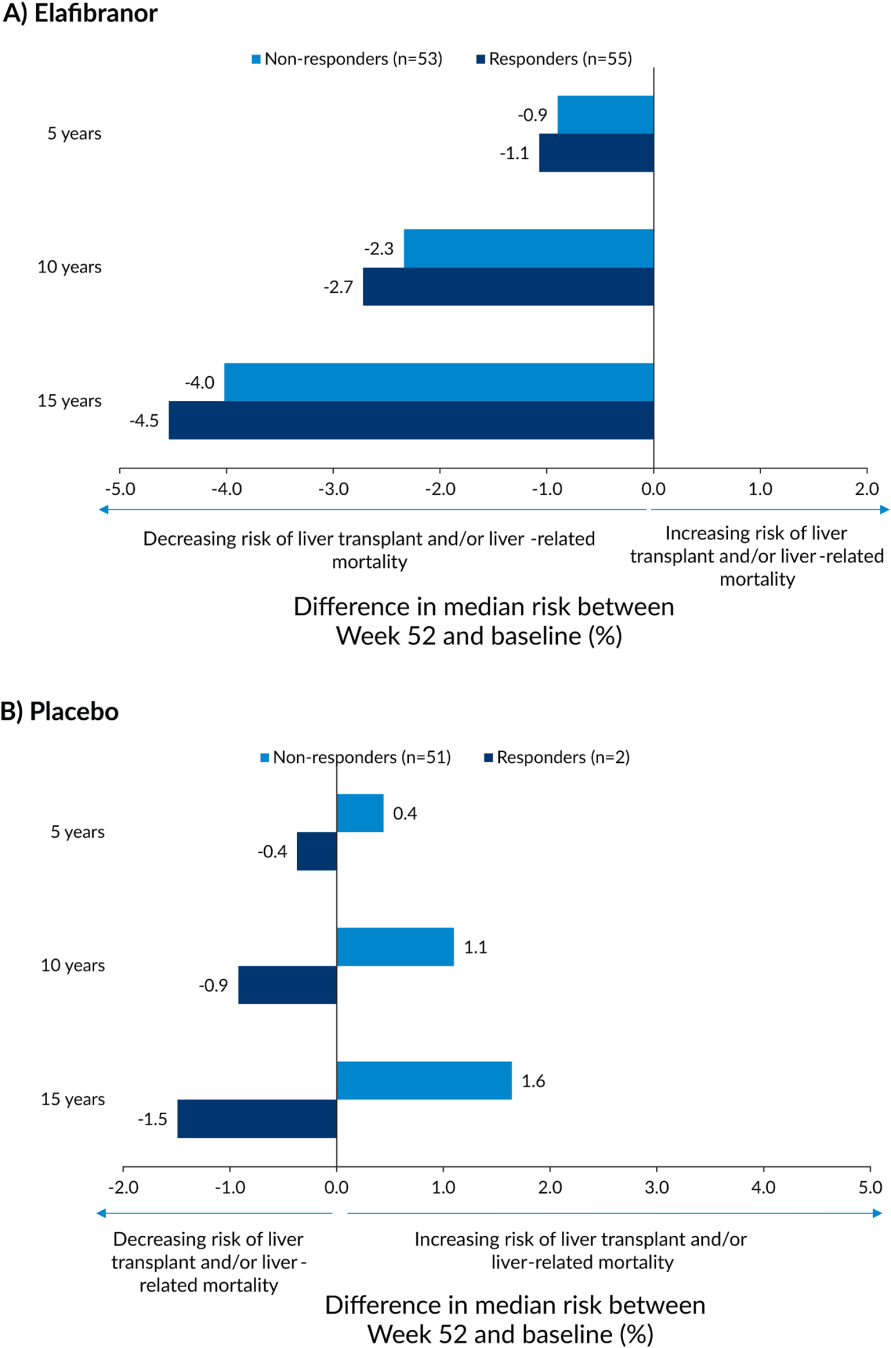
Risk change in estimated transplant‐free survival rates based on GLOBE score between Week 52 and baseline, stratified by achievement of biochemical response according to the primary endpoint^†^. ^†^Defined according to the POISE criteria (ALP < 1.67× ULN, with ≥ 15% reduction from baseline, and TB≤ULN). TB ULN value was 1.2 mg/dL in females and males; ALP ULN values were 104 U/L in females and 129 U/L in males. ALP: Alkaline phosphatase; TB: Total bilirubin; ULN: Upper limit of normal.

Regardless of biochemical response, patients receiving elafibranor had similar reductions in median risk at 5, 10, and 15 years (Figure 5A). For example, patients who met the primary endpoint while receiving elafibranor (*n* = 55) had a −4.5% reduction in median risk at 15 years, which was comparable to those who did not meet the primary endpoint (*n* = 53; −4.0%). Among patients receiving placebo, only those who met the primary endpoint (*n* = 2) had a reduction in median risk at 5, 10, and 15 years; patients who did not meet the primary endpoint (*n* = 51) had an increase in median risk (Figure 5B).

## 4 Discussion

Baseline ALP level distribution has shown variability across studies in PBC, irrespective of differences in disease presentation and severity [10, 11, 12]. ALP and TB are well‐established biochemical markers of cholestasis and disease progression in PBC [1, 2]. While biochemical response criteria (e.g., ALP level < 1.67× ULN, with a reduction of ≥ 15% from baseline, and TB≤ULN) are commonly used in clinical trials in PBC, the distribution of baseline values and the ULNs used may differ and influence the likelihood to meet such criteria. For example, in the ELATIVE trial, 39.1% of patients presented with a baseline ALP > 3× ULN (ULN: 104 U/L in females; 129 U/L in males) [10]. In contrast, patients in the treatment arm of the BEZURSO trial had a median baseline ALP of 2.3× ULN, and 20% presented with a baseline ALP > 3× ULN [13]. In the RESPONSE trial, patients in the treatment arm presented with a mean baseline ALP of 2.7× ULN, and 27.5% with a baseline ALP > 3× ULN [12, 14]. As shown in this analysis of ELATIVE data, patients with higher baseline ALP levels required proportionally greater reductions to meet the biochemical response threshold or achieve ALP normalization. These results also indicate that response rates can vary depending on baseline ALP values. Moreover, assigned ULN values are dependent on the particular laboratory used, which can influence overall response rates and therefore limit the comparability of drug efficacy across different trials [15, 16].

Treatment with elafibranor resulted in reductions in ALP levels across the baseline ALP subgroups, with the majority of patients having ALP decreases of 40.0% or more and TB≤ULN by Week 52. While percentage reductions in ALP across subgroups were in a comparable range, biochemical response rates according to the primary endpoint and ALP normalization were higher in the lowest baseline ALP subgroups compared with the highest baseline ALP subgroups because they were closer to the predefined thresholds of response. In patients with baseline ALP ≤ 2× ULN, 86.7% achieved biochemical response compared with 18.8% in patients with baseline ALP > 4× ULN. ALP normalization was achieved in 53.3% of patients with baseline ALP ≤ 2× ULN compared with 12.5% in patients with baseline ALP > 4× ULN.

While higher ALP baseline levels were generally associated with lower ALP normalization rates, normalization was observed across most baseline categories. Notably, two patients with baseline ALP > 4× ULN achieved ALP normalization by Week 52. However, no patients in the > 3–≤ 4× ULN subgroup achieved ALP normalization. These results support the efficacy of elafibranor, even in patients with substantially elevated baseline ALP. In this analysis, only 9 patients treated with elafibranor did not experience a reduction in ALP after 52 weeks of treatment.

Beyond consistent reductions in ALP, treatment with elafibranor was associated with an improvement in predicted transplant‐free survival over 15 years, as assessed by GLOBE. These findings are consistent with prior studies showing a near log‐linear relationship between ALP levels and transplant‐free survival, suggesting that any reduction in ALP confers prognostic benefit [4]. Moreover, among patients treated with elafibranor, those who did not meet the primary endpoint showed comparable reductions in risk to those who met the primary endpoint, according to the GLOBE score. This effect was not observed in the placebo group, suggesting that elafibranor is effective in reducing risk regardless of response classification. Additionally, the reduction in risk with elafibranor was relatively similar across the ALP subgroups. Together, these findings suggest that categorical definitions of biochemical response (i.e., dichotomous endpoints) may oversimplify or mask the true treatment benefits of elafibranor, or other therapies in PBC, and highlight the importance of continuing therapy in patients with incomplete biochemical response.

Altogether, assessments of treatment efficacy should account for key aspects of trial design, including the definition of biochemical endpoints, the ULN values for ALP, and the distribution of baseline values. Incorporating continuous outcome measures and predictive prognostic scores is also essential to capture the full extent of therapeutic benefit. This approach enables a more nuanced interpretation of treatment efficacy, and can also assist clinicians in setting realistic treatment goals, recognizing that meeting classical biochemical response criteria or ALP normalization may not be attainable for all patients, particularly within one year of treatment [17].

## 5 Conclusion

In the ELATIVE cohort, elafibranor demonstrated biochemical efficacy across the full spectrum of baseline ALP levels, supporting its broad therapeutic ability. While higher baseline ALP was associated with lower rates of biochemical response and ALP normalization, patients receiving elafibranor demonstrated consistent reductions in ALP levels across the baseline ALP subgroups. These reductions are expected to translate into improved clinical outcomes, as predicted by the increased chances of transplant‐free survival regardless of biochemical response, estimated using GLOBE scores. These results highlight that integrating additional considerations beyond dichotomous biochemical response rates can provide a more detailed understanding of a treatment's biochemical efficacy.

## Author Contributions

C.L., C.L.B., E.L., N.A., B.M., J.S., C.O.Z., K.V.K. substantial contributions to study conception and design; C.L., C.L.B., E.L., N.A., B.M., J.S., C.O.Z., K.V.K. substantial contributions to analysis and interpretation of the data; C.L., C.L.B., E.L., N.A., B.M., J.S., C.O.Z., K.V.K. drafting the article or reviewing it critically for important intellectual content; C.L., C.L.B., E.L., N.A., B.M., J.S., C.O.Z., K.V.K. final approval of the version of the article to be published.

## Funding

This secondary analysis and publication were sponsored by Ipsen. This study was sponsored by GENFIT.

## Ethics Statement

The trial was conducted in accordance with the International Council for Harmonisation Good Clinical Practice guidelines, applicable regulatory requirements and the principles of the Declaration of Helsinki.

## Consent

All patients provided written informed consent.

## Conflicts of Interest

C.L.: Received grants from Calliditas, CymaBay, Escient, Gilead, GlaxoSmithKline, Intercept, Ipsen, Kowa, Mirum, Target RWE and Zydus and is associate editor of Hepatology; C.L.B.: Received grants from Calliditas, ChemoMab, COUR, CymaBay, Gilead, GlaxoSmithKline, Hanmi, Intercept, Ipsen, Mirum, Novartis, Novo Nordisk, Pliant Therapeutics, Viking and Zydus; received consulting fees from AstraZeneca, ChemoMab, CymaBay, Esperion, GlaxoSmithKline, Ipsen, Intercept, Kezar, Mirum, NGM Bio, Pliant Therapeutics and RegCell; E.L.: Received grants from 89Bio Inc., AbbVie, Akero, Allergan, Alnylam, Amgen, Ascelia, Assembly Bio, AstraZeneca, Axcella Health, Biocryst, Bird Rock Bio Inc., Boehringer Ingelheim, Bristol Myers Squibb, Conatus, CymaBay, CytoDyn, DSM, Durect Corporation, Eli Lilly, Enanta, Enyo, Exalenz Bioscience, Galectin, Galmed, Genentech, GENFIT, Gilead, GlaxoSmithKline, Hanmi, Hightide, Intercept, Inventiva, Ipsen, Janssen, Laboratory for Advanced Medicine, Loxo Oncology, Madrigal, Merck, Metacrine, NGM Bio, NorthSea, Novartis, Novo Nordisk, Pfizer, Poxel, Roche, Sagimet, Synlogic, Takeda, Terns, Viking Therapeutics and Zydus; received consulting fees from Akero, Boehringer Ingelheim, Bristol Myers Squibb, Intercept, Metacrine, Novo Nordisk, Sagimet and Terns; received payment or honoraria from AbbVie, Gilead, Intercept and Madrigal; N.A.: Employee and shareholder of Ipsen; B.M.: Employee and shareholder of Ipsen; J.S.: Employee of Ipsen; C.O.Z.: Employee of Ipsen at the time of this study; K.V.K.: Received grants from Boston Scientific, Corcept, CymaBay, GENFIT, Gilead, GlaxoSmithKline, Hanmi, Intercept, Ipsen, Janssen, Madrigal, Mirum, Novo Nordisk, NGM Bio, Pfizer, Pliant Therapeutics, Terns, Viking, Zydus and 89bio Inc.; received royalties or licenses from UpToDate; received consulting fees from CymaBay, Enanta, GENFIT, Gilead, HighTide, Inipharm, Intercept, Ipsen, Madrigal, Mirum, NGM Bio, Pliant Therapeutics, Pfizer, Protagonist, Zydus and 89bio Inc.; received payment or honoraria from AbbVie, Gilead and Intercept; received payment for expert testimony from the US Department of Justice; participant on a Data Safety Monitoring Board or Advisory Board for CTI, Medpace, Labcorp and Worldwide Clinical Trials; stockholder in Inipharm; receipt of equipment, materials, drugs, medical writing, gifts or other services from Velacur.

## Supporting information


**Table S1:** Percentage and absolute reduction in ALP required to meet biochemical response and ALP normalization criteria for patients in each ALP baseline subgroup.
**Figure S1:** Risk change in estimated transplant‐free survival rates based on GLOBE score between Week 52 and baseline, stratified by baseline ALP level.
**Figure S2:** Risk change in estimated transplant‐free survival rates based on GLOBE score between Week 52 and baseline, stratified by ALP normalization†.

**Data S1:** CONSORT_2025.

## Data Availability

Qualified researchers with a valid research question may request anonymized patient‐level data by contacting an Ipsen representative. Further information on Ipsen's Data Sharing policy is available here: https://www.ipsen.com/science/clinical‐trials/clinical‐data‐transparency/.
