# Supplementary material for: Baseline Alkaline Phosphatase Impacts Response Rates in Primary Biliary Cholangitis: Exploring Response to Elafibranor in ELATIVE
Source: Liver Int. 2026 Apr 6;46(5):e70630. doi: 10.1111/liv.70630 (PMC13051278; doi:10.1111/liv.70630)
Supplement: Supplementary file 1 — Table S1: Percentage and absolute reduction in ALP required to meet biochemical response and ALP normalization criteria for patients in each ALP baseline subgroup. Figure S1: Risk change in estimated transplant‐free survival rates based on GLOBE score between Week 52 and baseline, stratified by baseline ALP level. Figure S2: Risk change in estimated transplant‐free survival rates based on GLOBE score between Week 52 and baseline, stratified by ALP normalization†. [file LIV-46-0-s002.docx]

**Baseline Alkaline Phosphatase Impacts Response Rates in Primary Biliary Cholangitis: Exploring Response to Elafibranor in ELATIVE^®^**

Cynthia Levy,^1,2^ Christopher L. Bowlus,^3^ Eric Lawitz,^4^ Nuno Antunes,^5^ Benjamin Miller,^5^ Jianfen Shu,^5^ Claudia O. Zein,^5*^ Kris V. Kowdley^6^

^1^Schiff Center for Liver Diseases, University of Miami, Miami, FL, USA; ^2^Division of Digestive Health and Liver Diseases, University of Miami School of Medicine, Miami, FL, USA; ^3^Division of Gastroenterology and Hepatology, UC Davis School of Medicine, Sacramento, CA, USA; ^4^The Texas Liver Institute, University of Texas Health, San Antonio, TX, USA; ^5^Ipsen, Cambridge, MA, USA; ^6^Liver Institute Northwest, Seattle, WA, USA

*Affiliation at the time of this analysis. Current affiliation: Eli Lilly and Company, Indianapolis, IN, USA

**Funding:** This secondary analysis and publication were sponsored by Ipsen. This study was sponsored by GENFIT.

**Trial registration:** NCT04526665

**Key words:** Alkaline phosphatase; biochemical markers; elafibranor; primary biliary cholangitis; prognosis

SUPPLEMENTARY MATERIALS

Supplementary Table S1. Percentage and absolute reduction in ALP required to meet biochemical response and ALP normalization criteria for patients in each ALP baseline subgroup

|  |  | **Baseline ALP subgroup** | | | | |
| --- | --- | --- | --- | --- | --- | --- |
|  |  | **≤2× ULN** | **>2–≤2.5× ULN** | **>2.5–≤3× ULN** | **>3–≤4× ULN** | **>4× ULN** |
| **Biochemical response** | Percentage reduction in ALP (%) | ≥15.0^†^–≤16.5 | >16.5–≤33.2 | >33.2–≤44.3 | >44.3–≤58.3 | >58.3 |
|  | Absolute reduction in ALP (U/L) |  |  |  |  |  |
|  | Female | ≥30.6−≤34.3 | >34.3–≤86.3 | >86.3–≤138.3 | >138.3–≤242.3 | >242.3 |
|  | Male | ≥38.0−≤42.6 | >42.6–≤107.1 | >107.1–≤171.6 | >171.6–≤300.6 | >300.6 |
| **ALP normalization** | Percentage reduction in ALP (%) | ≥40.1−≤50.0 | >50.0–≤60.0 | >60.0–≤66.7 | >66.7–≤75.0 | >75.0 |
|  | Absolute reduction in ALP (U/L) |  |  |  |  |  |
|  | Female | ≥69.7−≤104.0 | >104.0–≤156.0 | >156.0–≤208.0 | >208.0–≤312.0 | >312.0 |
|  | Male | ≥86.4−≤129.0 | >129.0–≤193.5 | >193.5–≤258.0 | >258.0–≤387.0 | >387.0 |

^†^A ≥15.0% reduction in ALP is required to meet the primary endpoint of the ELATIVE^®^ trial. ALP: alkaline phosphatase; ULN: upper limit of normal. 104 U/L (Female) and 129 U/L (Male) ULN values were used to generate these thresholds.

Supplementary Figure S1. Risk change in estimated transplant-free survival rates based on GLOBE score between Week 52 and baseline, stratified by baseline ALP level


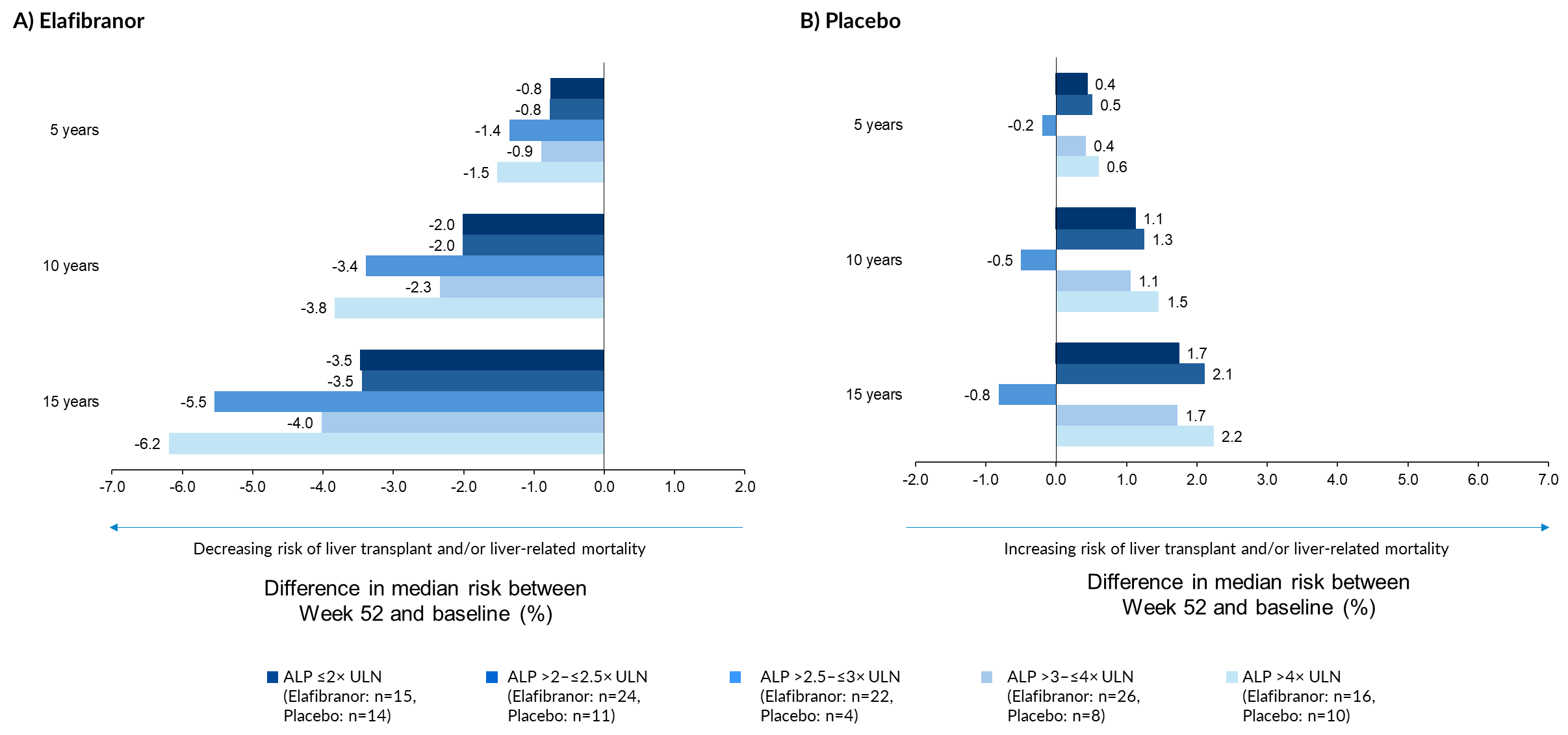


ALP: alkaline phosphatase; ULN: upper limit of normal.

Supplementary Figure S2. Risk change in estimated transplant-free survival rates based on GLOBE score between Week 52 and baseline, stratified by ALP normalization^†^


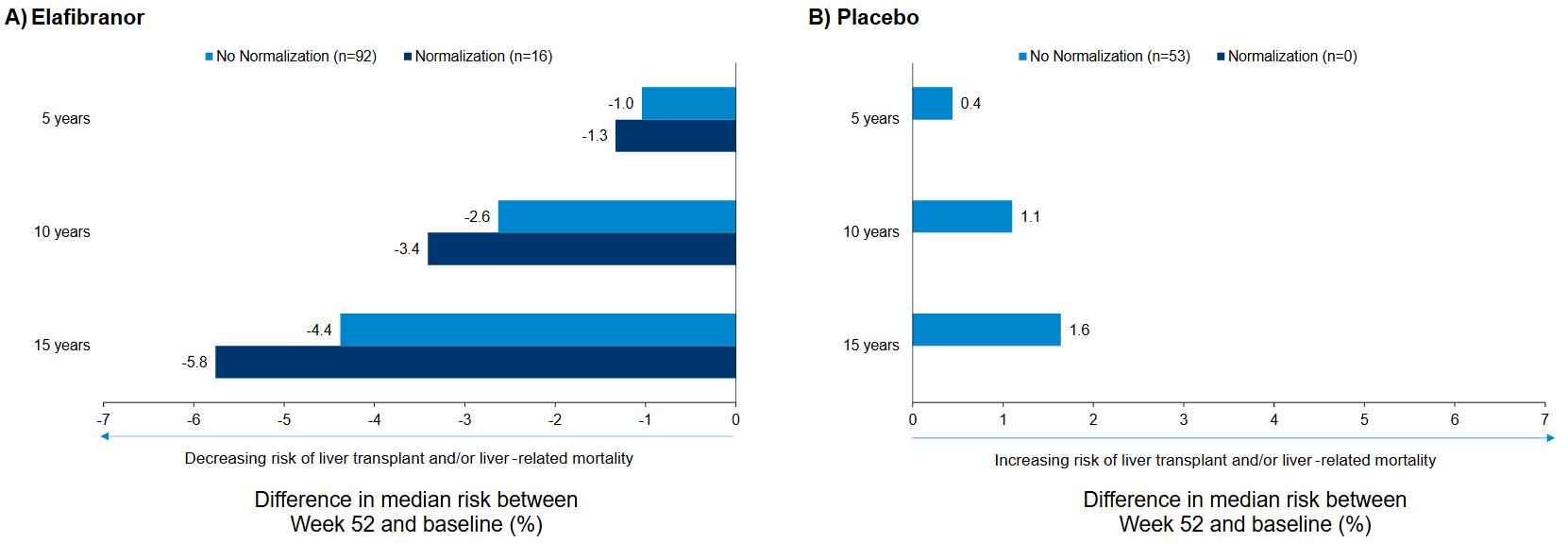


**^†^**Defined as ALP ≤ULN at Week 52. ALP ULN values were 104 U/L in females and 129 U/L in males. No patients receiving placebo achieved ALP normalization. ALP: alkaline phosphatase; ULN: upper limit of normal

Plain Language Summary

Elafibranor is a new treatment for PBC (primary biliary cholangitis), a rare liver disease that can become life-threatening. To see how someone with PBC is doing, doctors monitor substances in blood. When these substances reach specific target levels after treatment, it shows the medicine is working. However, people have different starting levels, which could affect target achievement. Relying on these targets alone can hide full treatment benefits or important health changes.

This research examined people with PBC who participated in the ELATIVE^®^ clinical trial after 1 year, grouped based on starting levels of a substance called ALP (alkaline phosphatase). We assessed how ALP starting levels influenced how well elafibranor worked compared with placebo (tablet with no medicine). More people with low starting ALP reached a target called ‘biochemical response’ than those with high starting ALP. However, despite different starting levels, everyone taking elafibranor showed similar improvements in ALP and several people taking elafibranor saw their levels return to normal. This did not happen for anyone taking placebo.

We also predicted the chances of needing a liver transplant over time after 1 year of treatment, and whether reaching ‘biochemical response’ affected this; people taking elafibranor had a reduced chance, whether or not they achieved the target.

Elafibranor was an effective treatment for PBC, but people with high starting ALP levels might not achieve biochemical response, even if they are benefiting from treatment. Therefore, it is important to consider overall changes in substances and other factors to understand if treatment is working.
